# Bioinformatics Analysis of Candidate Genes Related to Fat Deposition in Yaks at Different Energy Levels

**DOI:** 10.3390/cimb47060385

**Published:** 2025-05-22

**Authors:** Boxuan Yang, Xiaolin Luo, Xiangfei Zhang, Tianwu An, Qin Bai, Quan Sha, Hongwen Zhao

**Affiliations:** Sichuan Academy of Grassland Sciences, Chengdu 611731, China; ybx422830@163.com (B.Y.); luoxl2004@sina.com (X.L.); zxfsicau@foxmail.com (X.Z.); antianwu@126.com (T.A.); bq1278560523@163.com (Q.B.); 17360296361@163.com (Q.S.)

**Keywords:** fat deposition in yaks, differentially expressed genes, transcriptome sequencing, adipose cytokine signaling pathways, AMPK signaling pathway

## Abstract

Fat deposition is important for the growth and reproduction of yaks. To investigate the differentially expressed genes in muscle tissue and fat deposition in yaks at varying energy levels, 12 healthy adult yaks with similar body conditions were selected as research subjects. They were slaughtered after being reared at the following three different energy levels: low (L), medium (M), and high (H). The most extensive dorsal muscles were collected and screened for fat metabolism-related genes using Illumina for transcriptome sequencing. The results of transcriptome analysis showed that a total of 1430 differentially expressed genes were identified across the three groups of samples. Among these, 281 differentially expressed genes were identified between the high-energy group and the low-energy group; 365 differentially expressed genes were identified between the low-energy group and the medium-energy group; and 784 differentially expressed genes were identified between the medium-energy group and the high-energy group. GO and KEGG annotations indicated that across the three different energy levels the main enriched genes were found in the adipose cytokine signaling pathways, including, AMPK, the MAPK signaling pathway, and the PI3K-Akt signaling pathway. Both up- and down-regulation of *FGF-10* and *NR4A1* expression were found in fat deposition-related candidate genes; the seven up-regulated genes were *FGF-10*, *ACACB*, *DUSP1*, *c-FOS*, *NR4A1*, *RGS2*, and *FOXO1*, and the ten down-regulated genes were *LDLR*, *IRS2*, *FGF (FGF-10)*, *TRAF2*, *NR4A1*, *HSPB1*, *SGK1*, *MYL3*, *LEPR*, and *SLC2A1*. Two of the most common fat deposition genes, *FASN* and *PDK4*, were selected for q-PCR validation, along with ten candidate genes obtained from the transcriptome screening. The results showed that the expression trends of 12 genes in the three different energy level groups were consistent with those from transcriptome sequencing. This study revealed the complex transcriptome profiles of fat deposition in the muscle tissues of yaks at varying energy feeding levels and uncovered candidate genes involved in fat deposition.

## 1. Introduction

Yaks (Bos grunniens) possess unique physiological adaptation mechanisms to high-altitude ecological niches, particularly regarding fat deposition [1]. Fat, especially intramuscular fat (IMF), is a vital energy reservoir in the harsh conditions of highlands and significantly affects meat quality, which is crucial for domestic and export markets. The physiological mechanisms regulating fat deposition in yaks involve intricate interactions among genetics, environment, and nutrition [2], making it have an excellent texture, high protein, and low fat content [3,4]. The development of intramuscular fat (IMF), often referred to as intramuscular marbling, is a key factor that significantly impacts beef flavor and quality. A thorough understanding of the biological processes behind this trait is essential for selective breeding and optimizing feeding management to enhance beef quality [5]. Although research has reported fat deposition in yaks, particularly how dietary energy levels influence phenotypic characteristics such as IMF [6,7], additional research is required to describe the molecular foundation regulating these mechanisms. An understanding of fat metabolism at the molecular level in yaks is essential given that yaks are very important livestock in regions where local cattle breeds do not perform well due to strong climatic conditions. Current literature demonstrates that yaks possess unique metabolic adaptations that facilitate the effective utilization of energy obtained from forage found at high elevations [6,8]. Despite the understanding of the critical contribution of dietary energy to body composition and the growth performance of yaks, a substantial gap exists with respect to the transcriptomic mechanisms by which fat accumulation in muscular tissue in response to different levels of dietary energy is controlled.

Earlier research has emphasized the role of genetic factors, with a number of candidate genes, including fatty acid synthase (FASN), peroxisome proliferator-activated receptors (PPARs), and various members of signaling pathways, being implicated in lipid metabolism and accumulation [6,9]. For example, the role played by fatty acid synthase (FASN) in promoting the synthesis of fatty acids is emphasized, with empirical evidence pointing toward a correlation between its level of expression and intramuscular fat (IMF) content in various species [9]. However, when specifically considering yaks, the lack of transcriptomic research linking nutritional variation to gene-level regulation hinders an in-depth understanding of the underlying biological processes involved. Despite interesting phenotypic observations, the absence of integrative studies on how dietary macronutrient variation concurrently regulates gene expression in yaks, particularly for muscle lipogenesis, is an open avenue. RNA-sequencing (RNA-Seq) technology provides a useful tool to explore the genome-wide expression patterns of non-model organisms such as yaks and thus fill this knowledge gap. This throughput approach allows for a wide-ranging screening of gene expression diversity, which permits researchers to pick up slight changes throughout the transcriptome that are usually missed in conventional gene expression research [10].

The current study seeks to reveal the impact of nutritional intervention on transcriptome alteration regarding yak metabolism and acclimatization to high-altitude conditions. This study aims to reveal the particular genes and pathways implicated in IMF deposition and in the process understand general biological pathways mediating fat metabolism in yaks in response to different nutritional levels. By closing the gap between observed phenotypic diversity in yaks and genetic structure, this study consolidates knowledge of yak biology under high-altitude conditions in the realm of climate resilience and livestock sustainability. Moreover, this research is destined to streamline breed improvement programs, targeted to particular fat deposition traits, hence giving a long-term route to enhancing the productivity and livelihood of pastoral communities that are dependent on yak farming and where resilience in animal rearing is becoming increasingly essential.

## 2. Materials and Methods

### 2.1. Animal Materials and Feeding Management

Twelve healthy male yaks with the same genetic background and body weight (230.15 ± 13.00) kg were selected in Jiulong County, Ganzi Tibetan Autonomous Prefecture, Sichuan Province, China, and divided into three groups (low-, medium-, and high-energy levels), with four yaks in each group. Moreover, the yaks were approximately 36 months at the beginning of the experiment and 40 months at the time of slaughter, with a total fattening period of 115 days, which is within the typical fattening duration for yaks. According to the nutritional needs of fattening cattle in the “Beef Cattle Feeding Standard” (NY/T 815-2004) [11], the nutritional requirements are referenced for feeding. The three carbohydrate-structured dietary treatments used were low starch to neutral detergent fiber ratio (SN), middle SN, and high SN, corresponding to Group L, Group M, and Group H, with SN values of 0.39, 0.50, and 0.62, respectively. The main components of the concentrates were corn, wheat bran, canola meal, cottonseed meal, and soybean hulls, and the roughage was whole silage corn, wheat straw, and corn stover. The ration composition and nutrient level for this experiment are listed in Table 1. Nutrient analysis was performed according to the “Feed Analysis and Feed Quality Testing Techniques”. Before the feeding experiment, yaks were brought into the lot, ear-tagged, immunized, and dewormed as per the general husbandry of cattle. The animals were kept under environmental control, and the experiment was composed of 7 days of acclimatization followed by 115 days of the fattening trial. In this way, test diets were gradually introduced to animals by gradually replacing the proportion of test feed in the original diet during the transition period. During the experimental periods, the animals were fed with a total amount of TMR (total mixed ration) twice per day, respectively, at 08:00 and 16:00, and free access to water was monitored throughout and recorded. The yaks were slaughtered in equal numbers after fasting for 24 h, with 8 h of restricted access to water prior to killing at the end of the feeding period. Tissue samples were collected from the longest dorsal muscles of all of the yaks. Immediately, the samples were labeled, flash-frozen in liquid nitrogen, transported to the laboratory in a cryogenic state, and then stored at −80 °C for analyses.

All animal experimental procedures strictly adhered to the guidelines established by the Regional Ethics Committee for Animal Experimentation and complied with the care regulations approved by Animal Protection. The animal experiment of this research was approved by the Experimental Animal Ethics Committee of the Sichuan Academy of Grassland Sciences (Approval No. 20220012).

### 2.2. Main Instruments

The experiments were carried out with the assistance of advanced laboratory equipment for higher precision and reliability of the experiment results. A SCILOGEX D3024R centrifuge was used for the RNA extraction process (SCILOGEX, Rocky Hill, CT, USA). An analytikjena-qTOWER2.2 fluorescence quantitative PCR instrument (Analytik Jena AG, Jena, Germany) was used for the quantitative PCR test, while an analytikjena-Easycycler PCR instrument from Germany was used for conventional PCR analysis. Specific primers for target gene amplification were designed, and the exact copy number of the target gene was accurately determined through real-time monitoring by fluorescent dyes or probes. Ultra-micro nucleic acid and protein samples were quantitatively detected with a scandrop100 ultra-micro nucleic acid and protein assay system (Analytik Jena AG, Jena, Germany). Sample preparation and pipetting were performed using highly precise pipettes from Bio-Rad (Bio-Rad Laboratories, Hercules, CA, USA). During this experiment, consistency and accuracy of sample addition were maintained automatically with the help of the M1-type q-PCR automatic spiking instrument (Sichuan Kejin Instrument Co., Ltd., Chengdu, China).

### 2.3. Main Reagents and Consumables

Reverse transcription kit (TUREscript 1st Stand cDNA Synthesis Kit) (Aidlab Biotechnologies Co., Ltd., Beijing, China) and 2× SYBR^®^ Green premix (Dongsheng Biotech Co., Ltd., Guangdong, China); 10 μL lance head, 200 μL lance head, 1 mL lance head (GCS Scientific, LLC, Farmingdale, NY, USA), and 200 μL RNase-free PCR reaction tube (Axygen Scientific, Inc., Union City, CA, USA); 1.5 mL RNase-free EP tubes (GCS Scientific, LLC, Farmingdale, NY, USA), low white PCR reaction tubes (Bio-Rad; Bio-Rad Laboratories, Inc., Hercules, CA, USA), and optical sealing film (Bio-Rad). Both the gun tips and EP tubes are sterilized and dried for spare use before use.

### 2.4. Experimental Methods

#### 2.4.1. IMF Level Analysis

For accurate measurement, the Soxhlet extraction method was used to determine the content of IMF. First, the samples of muscle mince were placed on porcelain plates and kept at 65 °C to dry to a constant weight with fascia removed. The percentage difference between the weight before and after drying was determined to be the air-dried moisture content of the sample (W0). Then, the samples were ground into powder, and then circa 0.5 g of the sample was wrapped in filter paper bags (W1) for further drying in a drying cabinet at 102 °C to a constant weight (W2). The prepared filter bags were transferred into the Soxhlet extractor, where the addition of anhydrous ether was made for the fat extraction at 6–7 h with a speed of 6–8 cycles per hour. Filter bags were once more dried at 102 °C to a constant weight (W3).

The IMF content was calculated using the following formula:

$$\left(W2-\frac{W3}{W2}\right)\times \left(1-W0\right)\times 100\%$$

Data were analyzed with GraphPad Prism 8. Differences between the three energy-level groups were contrasted a using one-way ANOVA, and post hoc pairwise differences were adjusted using Bonferroni-adjusted post hoc testing. Statistical significance was set at *p* < 0.05.

#### 2.4.2. Sequencing Library Construction and Sequencing

The NanoDrop 2000 spectrophotometer was utilized to detect the purity and concentration of RNA, while the Agilent_2100/LabChip GX was used to assess the integrity of RNA for library construction accurately. After completing the library construction, the Qubit 3.0 fluorescence quantification instrument was used for preliminary quantification, with a required concentration of more than 1 ng/µL. Subsequently, the Qsep400 high-throughput analysis system was employed to accurately detect the insert fragments of the library once they met the necessary specifications. Following library construction, the Qubit 3.0 fluorescence quantifier was again used for preliminary quantification, ensuring a concentration greater than 1 ng/µL. The Qsep400 high-throughput analysis system was then used to detect the inserted library fragments. After the library quality control was confirmed, 12 samples from three energy level groups were sequenced in PE150 mode using the Illumina NovaSeq6000 sequencing platform.

#### 2.4.3. Functional Annotation and Enrichment Analysis of Differentially Expressed Genes and Their Verification via qPCR

In order to investigate the DEGs further, functional annotations and enrichment analyses were accomplished using the GO and KEGG databases. The same results from analyses indicated several important biological processes and pathways relevant to fat deposition and energy metabolism. Total RNA from muscle tissues was extracted with the Trizol method (Thermo Fisher Scientific, Inc.Waltham, MA, USA), and cDNA synthesis was performed using the Aidlab TUREscript 1st Strand cDNA Synthesis Kit (Aidlab Biotechnologies Co., Ltd., Beijing, China) to validate such results. The experimental verification of ten DEGs, including *FASN* and *PDK4*, was conducted using RT-qPCR with actin as the internal reference gene. The experiments were performed in a mixture containing SYBR^®^ Green Supermix (Dongsheng Biotech Co., Ltd., Guangzhou, China), primer, cDNA, and RNA-free water. Thermal cycling was programmed according to the following specifications: initial denaturation at 95 °C for 30 min, followed by 39 amplification cycles at 95 °C for 10 s and at 60 °C for 30 s. For gene expression, the 2^−△△Ct^ method was used for quantification and one-way ANOVA analysis was employed with the level of significance at *p* < 0.05 (Table 2).

## 3. Results and Analysis

### 3.1. Comparison of IMF in Yak Muscle with Different Energy Levels

**Figure 1:**
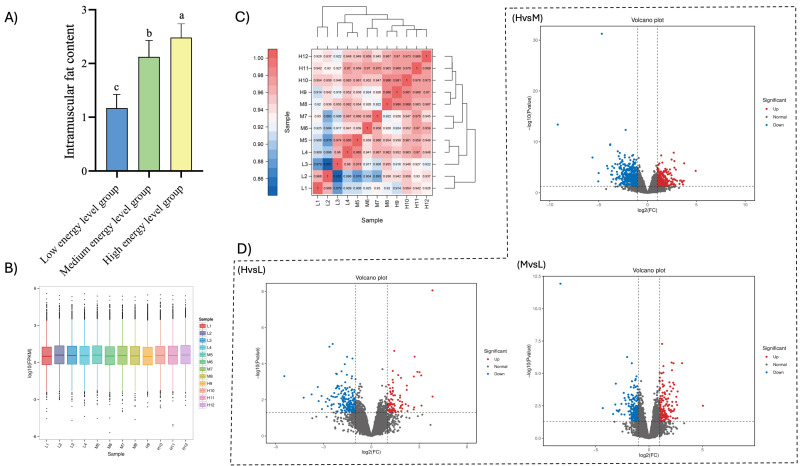
(**A**) Comparison of intramuscular fat (IMF) in yaks with different energy levels. Different letters indicate significant differences. (**B**) Box line plot of *FPKM* gene expression for each sample. The horizontal axis represents different samples, while the vertical axis indicates the logarithmic values of sample expression *FPKM*. The graph measures the expression level of each sample in terms of overall expression dispersion. (**C**) Heatmap of expression correlation between two samples. The value in each color block on the heatmap represents the correlation between the two samples on the horizontal and vertical axes corresponding to that block where the more significant the value, the higher the correlation is. (**D**) Differential expression volcano map. Blue dots represent down-regulated differentially expressed genes, red dots represent up-regulated differentially expressed genes, and gray dots represent non-differentially expressed genes. Different letters indicate statistically significant differences (Bonferroni post hoc test, *p* < 0.05).

As the energy level increased, the muscle IMF content rose significantly, with values of 1.17%, 2.12%, and 2.48% in the low-energy, medium-energy, and high-energy groups, respectively. Notably, the IMF content in the high-energy group was significantly elevated compared to both the low-energy and medium-energy groups (*p* < 0.05), and the medium-energy group had a considerably higher IMF content than the low-energy group (*p* < 0.05), as can be seen in Figure 1A.

### 3.2. Analysis of Sequencing Data Quality Control Assessment

After performing a series of quality control procedures, as described above, on 12 samples at three different energy levels, a total of 74.15 Gb of high-quality clean data were obtained, with 5.75 Gb of clean data for each sample. The percentage of Q30 bases for each sample was 91.69%, as shown in Table 3. Typically, the expression levels of protein-coding genes sequenced to FPKM (fragments per kilobase of transcript per million mapped reads) values span six orders of magnitude from 10^−2^ to 10^4^ [12], with most genes concentrated in the range of 0.1–10. A box plot of gene expression in each sample allows us to view the degree of dispersion of gene expression levels and also enables us to visually compare the overall level of gene expression across different samples. The results of this study indicated that the median, interquartile range, and gene expression distributions were similar within the same group, suggesting that the reproducibility of parallel samples in the same group was high, as detailed in Figure 1B. The correlation analysis of the sequencing data following the quality control demonstrated that the duplicate samples in the same group were highly similar, as shown in Figure 1C. The results from both the sample box-and-line plot and the correlation analysis indicate that the transcriptomic data from this study are of high quality and suitable for further analysis.

### 3.3. Analysis of Differentially Expressed Genes

**Figure 2:**
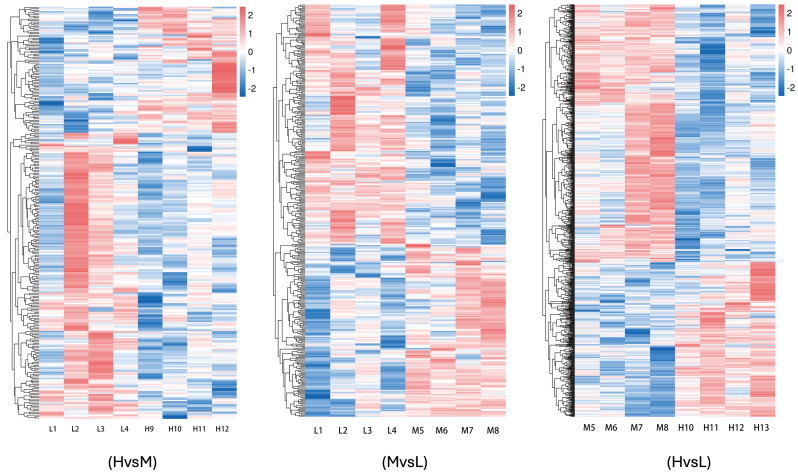
Clustering map of differentially expressed genes. The horizontal coordinates represent the sample names and the clustering results of the samples, while the vertical coordinates represent the differentially expressed genes and their clustering results. Different columns in the graph correspond to different samples and different rows correspond to different genes. The colors indicate the expression level log10 of the genes in the samples.

A total of 1430 differentially expressed genes were identified in the three groups of samples in this study, including 532 up-regulated genes and 898 down-regulated genes. Among them, there were 281 differentially expressed genes between groups H and L, comprising 86 up-regulated genes and 195 down-regulated genes. Between groups L and M, there were 365 differentially expressed genes, including 153 up-regulated genes and 212 down-regulated genes. Between groups M and H, a total of 784 differentially expressed genes, consisting of 293 up-regulated genes and 491 down-regulated genes, were identified. The results of hierarchical clustering analysis, as illustrated in the differentially expressed gene clustering heatmap, demonstrated that the four biological replicates in each of the three energy level groups were highly clustered, indicating a strong correlation between the samples. This further confirms the accuracy and reliability of the sequencing data (Figure 1D and Figure 2).

### 3.4. Differential GO Functional Annotation and KEGG Pathway Enrichment Analysis

**Figure 3:**
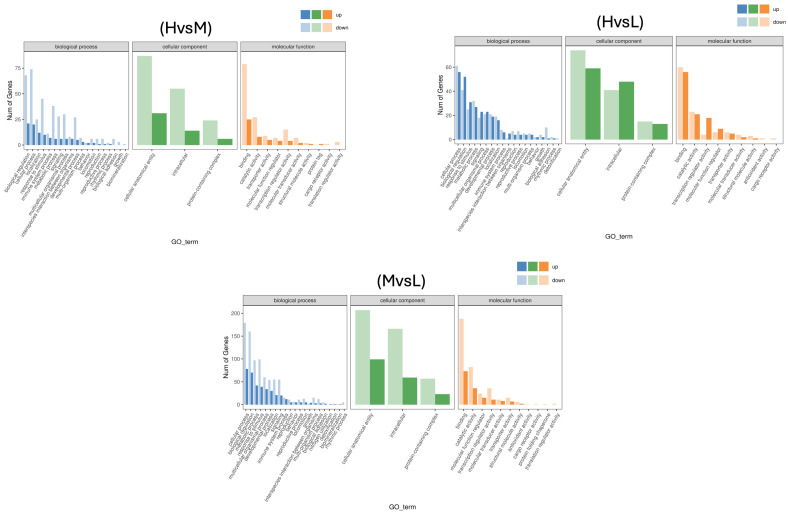
Classification statistics of GO annotations for differentially expressed genes. The horizontal axis represents the GO classifications, the vertical axis indicates the number of genes, and different colors denote the primary classifications to which they belong.

To further visualize the functional entries related to differential gene fat deposition, the differentially expressed genes were classified and counted at the secondary classification level of the GO database. A total of 32 entries were annotated between the H and L groups, encompassing 152 differentially expressed genes, with 38 and 114 involved in up- and down-regulated expression, respectively. A total of 35 entries were annotated between the H and M groups, encompassing 409 differentially expressed genes, with 136 and 273 involved in up- and down-regulated expression, respectively. A total of 31 entries were annotated between the M and L groups, encompassing 173 differentially expressed genes, with 73 and 100 involved in up- and down-regulated expression, respectively. They are mainly co-enriched in biological regulation, cellular processes, metabolic processes, cellular, anatomical entities, and intracellular, binding, and catalytic activity (Figure 3).

The KEGG pathway enrichment analysis revealed that the top 20 significant KEGG pathways were selected for presentation in each group, with a smaller q value indicating greater significance. Differentially expressed genes were significantly enriched in a total of 165 signaling pathways. The most significant enrichment among the first twenty pathways included bile secretion, the TNF signaling pathway, cytokine–cytokine receptor interaction, and the adipocytokine signaling pathway. A total of 92 genes were enriched, consisting of 25 up-regulated and 67 down-regulated genes. Pathways and genes associated with fat deposition included bile secretion, the TNF signaling pathway, the adipocytokine signaling pathway, and the regulation of the actin cytoskeleton. In groups H and M, the differentially expressed genes were enriched in a total of 273 signaling pathways and the top 20 pathways were selected. These indicated that the MAPK signaling pathway, adrenergic signaling in cardiomyocytes, the HIF-1 signaling pathway, nitrogen metabolism, arginine, and proline metabolism were the most significantly enriched. A total of 222 genes were enriched, including 79 up-regulated and 143 down-regulated. Among the pathways and genes related to fat deposition were the MAPK signaling pathway, nitrogen metabolism, arginine and proline metabolism pathway, the adipocytokine signaling pathway, adrenergic signaling in cardiomyocytes, and the PI3K-Akt signaling pathway.

**Figure 4:**
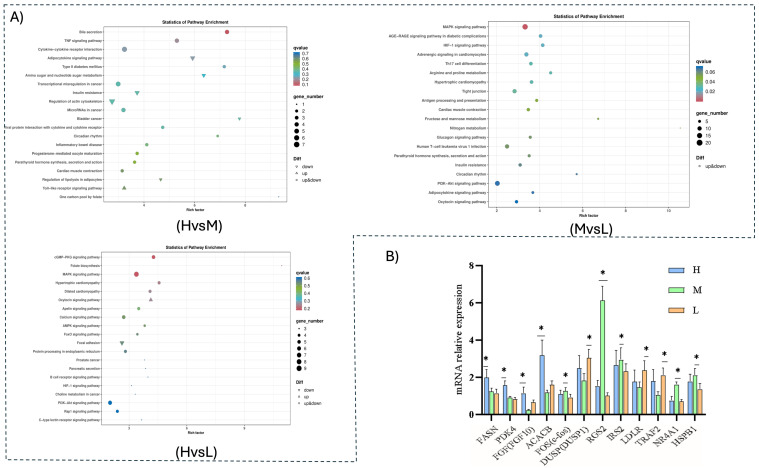
(**A**) Bubble map of KEGG enrichment of differentially expressed genes. Each circle in the figure represents a KEGG pathway; the vertical coordinate indicates the pathway name and the horizontal coordinate represents the rich factor, which reflects the ratio of the proportion of genes annotated to a pathway among the differentially expressed genes to the proportion of genes annotated to that pathway among all genes. (**B**) RT-qPCR validation of differentially expressed genes. “*” indicate statistically significant differences (Bonferroni post hoc test, *p* < 0.05).

Differentially expressed genes were enriched in groups M and L in 209 pathways. The top twenty pathways, which were most significantly enriched, included the cGMP-PKG signaling pathway, folate biosynthesis, the MAPK signaling pathway, the AMPK signaling pathway, and the PI3K-Akt signaling pathway. A total of 98 genes were identified, including 47 that were up-regulated and 51 that were down-regulated. Among the pathways and genes related to fat deposition were the MAPK signaling pathway, the AMPK signaling pathway, and cGMP-PKG signaling (Figure 4).

### 3.5. Related Functional Genes Screening and Analysis

Based on the enrichment of GO and KEGG pathways, as shown in Table 4, the top twenty pathways were selected from each group. The study results indicated that these pathways were primarily enriched in the adipocytokine signaling pathway, the P13K signaling pathway, the MAPK signaling pathway, and the cGMP-PKG signaling pathway, along with other genes related to fat deposition. In the H and L groups, in addition to LDLR which is a down-regulated gene in bile secretion, IRS2, which is a down-regulated gene in the adipocytokine signaling pathway, and FGF, which is involved the regulation of the actin cytoskeleton, were associated with fat deposition, as well as MYL3, which is a down-regulated gene in the TNF signaling pathway.

FGF, an up-regulated gene in the MAPK signaling pathway, and *TRAF2*, *NR4A1*, and *HSPB1*, down-regulated genes, were associated with fat deposition in the H and M groups. GADD45 (during muscle growth) and FLNA (multifunctional actin-binding proteins) were identified as being associated with muscle. The adipocytokine signaling pathway is linked to the fat deposition pathway, showing up- and down-regulated genes in the AMPK signaling pathway. *ACACB* is the up-regulated gene associated with fat deposition and *SLC2A1* is the down-regulated gene. The PI3K-Akt signaling pathway includes the up-regulated gene *FGF* and the down-regulated genes *SGK1*, *CREB5*, and *NR4A1*, each of which are associated with fat deposition. Additionally, the EIF4E gene was found to be associated with muscle traits. In the arginine and proline metabolic pathways, up-regulation of *MAOA* was related to reproductive traits, whilst down-regulation of the gene *MYL3* in adrenergic signaling in cardiomyocytes was linked with fat deposition, *TNNC1*, *TPM3*, *MYL2*, and *MYH6*-7 were found to be associated with muscle quality, and *CREM* was identified as being related to the regulation of reproduction.

In groups M and L, the up-regulated genes *DUSP*, *FOS*, and *NR4A1* along with the down-regulated gene FGF in the MAPK signaling pathway were associated with fat deposition. In the cGMP-PKG signaling pathway, the up-regulated gene *RGS2* was linked to fat deposition while the down-regulated gene *ATP2B* was found to be associated with reproductive performance and calcium enrichment. *NR4A1*, an up-regulated gene in the PI3K-Akt signaling pathway, and FGF, a down-regulated gene, were associated with fat deposition, and IGH was found to be related to meaty traits. In addition, *FOXO1*, an up-regulated gene in the AMPK signaling pathway, and *LEPR*, a down-regulated gene, were associated with fat deposition. The down-regulated gene ERBB2 in the focal adhesion signaling pathway was identified as being involved in negative muscle regulation during cellular processes, while the *SPP1* and *PDGFRA* genes were associated with the regulation of muscle quality and reproductive traits.

### 3.6. qRT-PCR

To verify the accuracy of the RNA-Seq results, 12 genes, including *FASN*, *PDK4*, and *FGF* (*FGF10*), were randomly selected for qPCR validation. By comparing the change patterns and significance of differences among the selected differential genes, the results showed that the expression trends of the 12 genes across three different energy level groups were consistent with both the earlier findings and the RNA-Seq results, thereby confirming the accuracy and reliability of the RNA-Seq data (Figure 4B).

## 4. Discussion

The investigation of the impact of dietary energy on intramuscular fat (IMF) deposition in yaks provides a meaningful perspective on adaptive metabolic responses to different energy intakes. The differences in gene expression profiles between groups of three energy levels (low, medium, and high) reveal the physiological adaptations employed by yaks to achieve optimal energy efficiency and fat deposition. The present discourse brings together the results of RNA-Seq and qPCR analysis, highlighting the importance of alterations in key metabolic processes, including the AMPK, MAPK, and PI3K-Akt signaling pathways, and the resulting effect on the quality of yak meat and breeding strategies. In this research, we found notable regulation in intramuscular fat (IMF) content with noteworthy alteration of transcriptomic profiles under three energy levels (low, medium, and high). As yaks were transitioning from low (L) to moderate (M) and high (H) diet levels a significant rise in intramuscular fat (IMF) was found, demonstrating the direct connection between energy consumption and adipose tissue accumulation. Such a finding corresponds with other research on ruminant animals where higher levels of energy support fat accumulation by lipogenic gene activation and mechanisms involved in fatty acid metabolism [6,13]. One example of such an occurrence is the up-regulation of fatty acid synthase (FASN) and acetyl-CoA carboxylase (ACACB) under high-energy conditions, signaling a metabolic shift towards increased lipogenesis. While FASN plays the critical role of converting excess energy into fatty acids, ACACB is involved in the production of malonyl-CoA which is an important precursor for fatty acid synthesis [12,13,14]. The high-energy diet might activate these pathways as a mechanism for the control of excess energy, thus inducing fat deposition for the purpose of buffering in conditions of low energy availability and suggesting an evolutionary adaptation to hostile environmental conditions.

We found 1430 differentially expressed genes (DEGs), suggesting considerable interaction between energy consumption and gene regulation for fat metabolism in yak longissimus dorsi muscle tissues. The pathways under consideration, i.e., AMPK, MAPK, and PI3K-Akt, are essential to the control of metabolism and have been documented in yaks as well as in other domestic species, thus providing a foundation for understanding the molecular adaptations taking place in yak physiology in adaptation to varied nutritional diets [15,16]. The AMPK signaling pathway acts as an intracellular energy sensor and is found to promote lipid oxidation and inhibit de novo lipogenesis simultaneously. Thus, its role is indispensable in energy deficit and surplus states [16]. Importantly, the up-regulated expression of genes participating in the AMPK pathway coupled with the elevated intramuscular fat (IMF) content in high-energy diets suggests a possible compensatory mechanism through which yak muscle tissues can promote fat accumulation to sustain future energy demands. This aligns with findings in other livestock, whereby AMPK activation is negatively associated with IMF deposition, and demonstrates a conserved biological theme across species [15,17]. In different situations, enzyme inhibition associated with fatty acid synthesis after high-energy feeding indicates a change in metabolism to fat storage, which may mirror evolutionary adaptations of yaks that allow for energy conservation in drought or nutrition deficiency [18,19]. The other pathway of interest, PI3K-Akt, is vital to cell metabolism and growth by mediating insulin signaling and lipogenic processes. Our study’s expression data indicated that genes included in PI3K-Akt had significant changes across varying levels of energy, thereby confirming its established role of promoting adipogenesis through up-regulating principal adipogenic transcription factors such as PPARγ [16,17]. While studies have a tendency to show that PI3K-Akt facilitates lipid storage in adipocytes, the expression in yak muscle may suggest a shift in its role whereby certain Akt isoforms may play varying roles in muscle metabolism compared to those in adipose tissue to address specific physiological needs [20]. Moreover, in comparison with other livestock studies, yaks share both similarities and differences in the patterns of metabolic regulation. For example, the up-regulation of genes involved in lipid synthesis under high-energy intake is comparable to findings in cattle and sheep [21]. However, the specific down-regulation of some nuclear receptors, like NR4A1, suggests a special regulatory mechanism that might not be so easily detectable in non-adapted species. This suggests that although there is some similarity in gene function, yaks possess distinct regulatory mechanisms indicative of evolutionary adaptations to their respective ecological niches [22].

The MAPK signaling pathway, especially within the framework of adipocyte proliferation and differentiation, constitutes a key regulatory function in fat accumulation [23,24]. DEGs that belong to this pathway in yaks point toward a robust response to nutritional signals from the outside, regulating the transcriptional landscape to facilitate enhanced capacity for fat storage, particularly under high-energy-intake conditions [25,26]. This dynamic interaction of regulatory pathways calls for a more profound comprehension of metabolic plasticity, or a new understanding of how yaks regulate fatty acid storage and use that is responsible for maintaining homeostasis under varying conditions [6,21,27]. The complex interplay of these pathways generates intriguing questions regarding adaptive metabolic responses in yaks. For example, it was surprising to observe that NR4A1, a recognized modulator of lipid metabolism, exhibited down-regulation in the context of high-energy diets, notwithstanding its involvement in facilitating fatty acid oxidation and affecting multiple metabolic pathways [28]. This result may imply an adaptation mechanism by which decreased NR4A1 activity under high-energy conditions strives to equilibrate lipid accumulation and reduce the risk of lipotoxicity, a notion that has also been observed in research with other livestock [29].

The explanation of the IMF accumulation, in combination with the molecular findings, points to an adaptively adjusted response to the specific yak environment. As ruminants which are regularly subjected to variable nutritional landscapes, yaks must have evolved systems to optimize energy storage when they have plenty of food while still having the capacity to mobilize fat reserves during times of food scarcity [30,31]. The rises in fat deposition observed in our experiment are not only consistent with elevated dietary energy intake but also call into question meat quality. The effect of intramuscular fat on the flavor, tenderness, and juiciness of meat points to important implications for yak breeding and meat production systems. Together, these results uncover a complex regulatory environment governing fat deposition in yaks across different energy states, foregrounding the theme of metabolic flexibility in livestock populations. Expression profiles of genes including FASN, PDK4, and more tell a story of how energy level changes influence underlying physiological mechanisms, connecting molecular biology and real-world applications to breeding programs aiming to enhance meat quality and resilience to nutritional variations [15,21].

The findings of this study have implications beyond fundamental knowledge of metabolism; breeding yaks for optimal fat deposition according to genetic information can be expected to make a great contribution to improving the meat quality and thereby influencing the economic feasibility of yak farming. Furthermore, these findings have important potential to improve adaptive characteristics in yaks so that they are more resilient to climatic and environmental variations, thereby facilitating sustainable agriculture in areas where yaks play an important role in local economies and cultural contexts.

## 5. Conclusions

The gene expression analysis associated with adipose tissue deposition in yaks exposed to different dietary energy levels offers proof of the key metabolic reactions mirroring the adaptation and tolerance of the species to environmental stress. The coordinative role between the up-regulated lipogenic genes and down-regulated proteolytic markers indicates a well-regulated strategy to balance energy storage and metabolic health. These results hold significant implications for planning yak breeding programs and nutrition management, with a focus on optimizing diets not only for growth performance but also for better meat quality. This will influence the sustainability and efficiency of yak husbandry systems under unfavorable environments.

## Figures and Tables

**Table 1:** Composition and nutrient levels of diets (DM basis: %).

Items	Groups		
	L	M	H
Ingredients			
Corn	15.42	20.54	25.60
Wheat bran	13.22	7.18	2.22
Rapeseed meal	2.94	4.49	4.30
Cottonseed meal	7.58	7.00	7.50
Soybean hull	4.36	4.31	3.90
Yeast	0.15	0.15	0.15
Fatty acid calcium	1.06	1.06	1.06
Puffing urea	0.26	0.26	0.26
CaCo_3_	0.28	0.28	0.28
NaHCO_3_	1.11	1.11	1.11
NaCl	0.84	0.84	0.84
Premix ^(1)^	2.78	2.78	2.78
Whole corn silage	20.00	25.00	30.00
Wheat straw	20.00	15.00	7.50
Commn straw	10.00	10.00	12.50
Total	100.00	100.00	100.00
Nutrient levels ^(2)^			
DM/%	76.98	73.52	70.02
GE/(MI/kg)	18.4	18.46	18.51
CP/%	13.32	13.33	13.34
NDF/%	47.11	44.07	41.02
ADF/%	27.95	26.57	24.81
Ca/%	0.87	0.86	0.84
P/%	0.48	0.45	0.42
Starch/%	19.20	22.50	26.07
EE/%	3.36	3.46	3.57
SN	0.41	0.51	0.64

(1): The premix provided the following per kg of diet: vitamin A (VA), 2,100,000 IU; vitamin D3 (VD3), 800,000 IU; vitamin E (VE), 21,000 IU; copper (Cu), 6.2 g; manganese (Mn), 9.54 g; zinc (Zn), 12.42 g; iodine (I), 210 mg; selenium (Se), 180 mg; cobalt (Co), 75 mg. (2): GE (gross energy) was calculated according to the Chinese Beef Cattle Feeding Standards (NY/T 815-2004), while all other nutrient values are directly measured.

**Table 2:** The following primers were used in the study.

Gene Number	Gene Name	Primer Sequence (5′→3′)	Annealing Temperature
Reference genes	*Actin*-F	CGTGCGTGACATCAAAGAGAA	60
	*Actin*-R	AACCGCTCGTTGCCAATAGT	
1	*FASN*-F	CCACTTCCCACTGGAACAAGACAA	60
	*FASN*-R	GGAGGCGTAATAGATGGTGCAGAG	
2	*PDK4*-F	GGCTGCTTCCTGAACGTTTG	60
	*PDK4*-R	TCCGGTCACATCAGTGTTCG	
3	*FGF(FGF10)*-F	CTGAAGGAGAGGATAGAGGAA	60
	*FGF(FGF10)*-R	TGTACCACCATCGGAAGA	
4	*ACACB*-F	GCTTAGTTCCCTCGTTAGTAG	61
	*ACACB*-R	TCTTGCCTCTCTTCTCTGT	
5	*FOS(c-fos)*-F	CCAATCTGCTGAAGGAGAA	61
	*FOS(c-fos)*-R	CAAGAGAAGCCACAGACA	
6	*DUSP(DUSP2)*-F	GACAACAACGACAACAGC	62
	*DUSP(DUSP2)*-R	AGGTACAGGTAGGGCAAG	
7	*RGS2*-F	CAGGAAACATCACTCAGAACTA	60
	*RGS2*-R	TCTCTCTGTGCTCCATCA	
8	*IRS2*-F	GCCAGCATCGACTTCTTG	62
	*IRS2*-R	TGAGCGTCTTCTTCCCAT	
9	*LDLR*-F	CACAGGCTCAGACATCAG	61
	*LDLR*-R	AGTCCTCTCACACCAGTT	
10	*TRAF2*-F	GAGAACATCGTCTGTGTG	61
	*TRAF2*-R	GATCCTGTCTTGGTCCAG	
11	*NR4A1*-F	AATACAGGAAGGAAGAGGTG	61
	*NR4A1*-R	GAAGAGAGCAAGGAGGAG	
12	*HSPB1*-F	ATTTCCCGTTGCTTCACT	60
	*HSPB1*-R	GGACAGAGAGGAGGAGAC	

**Table 3:** Sequencing data statics.

Sample Number	Clean Reads	Clean Bases	GC Content	≥Q20 (%)	≥Q30 (%)
L1	20,935,047	6,267,647,694	52.87	97.21	92.60
L2	20,315,896	6,081,273,212	52.81	97.34	92.88
L3	2,0276,719	6,069,683,192	52.27	96.92	92.00
L4	21,768,354	6,515,945,116	52.01	96.74	91.69
M5	19,477,561	5,830,404,158	51.25	97.16	92.50
M6	21,247,585	6,360,558,784	51.80	97.31	92.82
M7	19,550,304	5,852,448,380	52.40	96.95	92.10
M8	20,332,295	6,086,162,254	52.05	97.47	93.21
H9	22,479,971	6,728,774,426	51.17	97.27	92.76
H10	19,197,776	5,746,377,886	53.00	97.15	92.52
H11	20,689,299	6,192,793,088	51.94	97.25	92.66
H12	21,452,857	6,420,292,678	52.51	96.92	92.08

Note: L1-4 are four replicates for samples in the low-energy-level group; M5–8 are four replicates for samples in the medium-energy-level group; H9–12 are four replicates for samples in the high-energy-level group; ≥Q20%: percentage of bases with a clean data mass value greater than or equal to 20; and ≥Q30%: percentage of bases with a clean data mass value greater than or equal to 30.

**Table 4:** KEGG terms related to lipid metabolisms.

Groups	Terms	Total Genes	*p* Value	Related Genes
**HVSL**	Bile secretion	5	0.0006	Down: *LDLR*
	Adipocytokine signaling pathway	4	0.0046	Down: *IRS2*
	Regulation of actin cytoskeleton	6	0.0682	Down: *FGF*
**HVSM**	MAPK signaling pathway	20	2.33 × 10^−6^	Up: *FGF*Down: *TRAF2*, *NR4A1*, *HSPB1*
	Adipocytokine signaling pathway	6	0.0056	Up: *ACACB*Down: *SLC2A1*
	PI3K-Akt signaling pathway	16	0.0056	Up: *FGF*Down: *SGK1*, *NR4A1*
**MVSL**	MAPK signaling pathway	9	0.0071	Up: *DUSP*, c-*FOS*, *NR4A1*Down: *FGF*
	cGMP-PKG signaling pathway	6	0.0028	Up: *RGS2*
	PI3K-Akt signaling pathway	7	0.0595	Up: *NR4A1*Down: *FGF*

## Data Availability

The original contributions presented in this study are included in the article. Further inquiries can be directed to the corresponding author.
